# Shaping the olfactory map: cell type-specific activity patterns guide circuit formation

**DOI:** 10.3389/fncir.2024.1409680

**Published:** 2024-05-27

**Authors:** Ai Nakashima, Haruki Takeuchi

**Affiliations:** ^1^Laboratory of Chemical Pharmacology, Graduate School of Pharmaceutical Sciences, The University of Tokyo, Tokyo, Japan; ^2^Department of Biophysics and Biochemistry, Graduate School of Science, The University of Tokyo, Tokyo, Japan

**Keywords:** olfactory map, neural activity, gene expression, odorant receptor, neural development

## Abstract

The brain constructs spatially organized sensory maps to represent sensory information. The formation of sensory maps has traditionally been thought to depend on synchronous neuronal activity. However, recent evidence from the olfactory system suggests that cell type-specific temporal patterns of spontaneous activity play an instructive role in shaping the olfactory glomerular map. These findings challenge traditional views and highlight the importance of investigating the spatiotemporal dynamics of neural activity to understand the development of complex neural circuits. This review discusses the implications of new findings in the olfactory system and outlines future research directions.

## 1 Introduction

Sensory information is represented in the brain as spatially organized activity patterns, commonly referred to as sensory or topographic maps (Luo and Flanagan, 2007; Petersen, 2007; Mori and Sakano, 2011; Pumo et al., 2022). The formation of precise sensory maps depends on the ordered projection of neurons, a process that is initially dictated by genetic programming and later fine-tuned through neural activity-dependent mechanisms. In the field of developmental neuroscience, numerous studies have suggested that correlated neural activity drives sensory map refinement (Katz and Shatz, 1996; Kirkby et al., 2013; Ackman and Crair, 2014; Pan and Monje, 2020; Martini et al., 2021). For example, in the developing visual system, spatially correlated spontaneous activity propagates in a wave-like manner across the retina (Galli and Maffei, 1988; Meister et al., 1991; Wong et al., 1993). In the somatosensory system, patchwork-like synchronized firing patterns corresponding to barrel maps are observed in the somatosensory cortex (Mizuno et al., 2018; Martini et al., 2021). In contrast, spontaneous activity in the primary olfactory system has been reported to lack spatiotemporal correlation, which contradicts the predictions of Hebb’s theory. A recent study suggests that cell type-specific temporal activity patterns may play an instructive role in the development of the olfactory map (Nakashima et al., 2019, 2021). This review summarizes the current understanding of the emerging evidence for olfactory circuit formation that appears to be guided independently of Hebbian plasticity rules.

## 2 Development of the olfactory glomerular map

**Figure 1:**
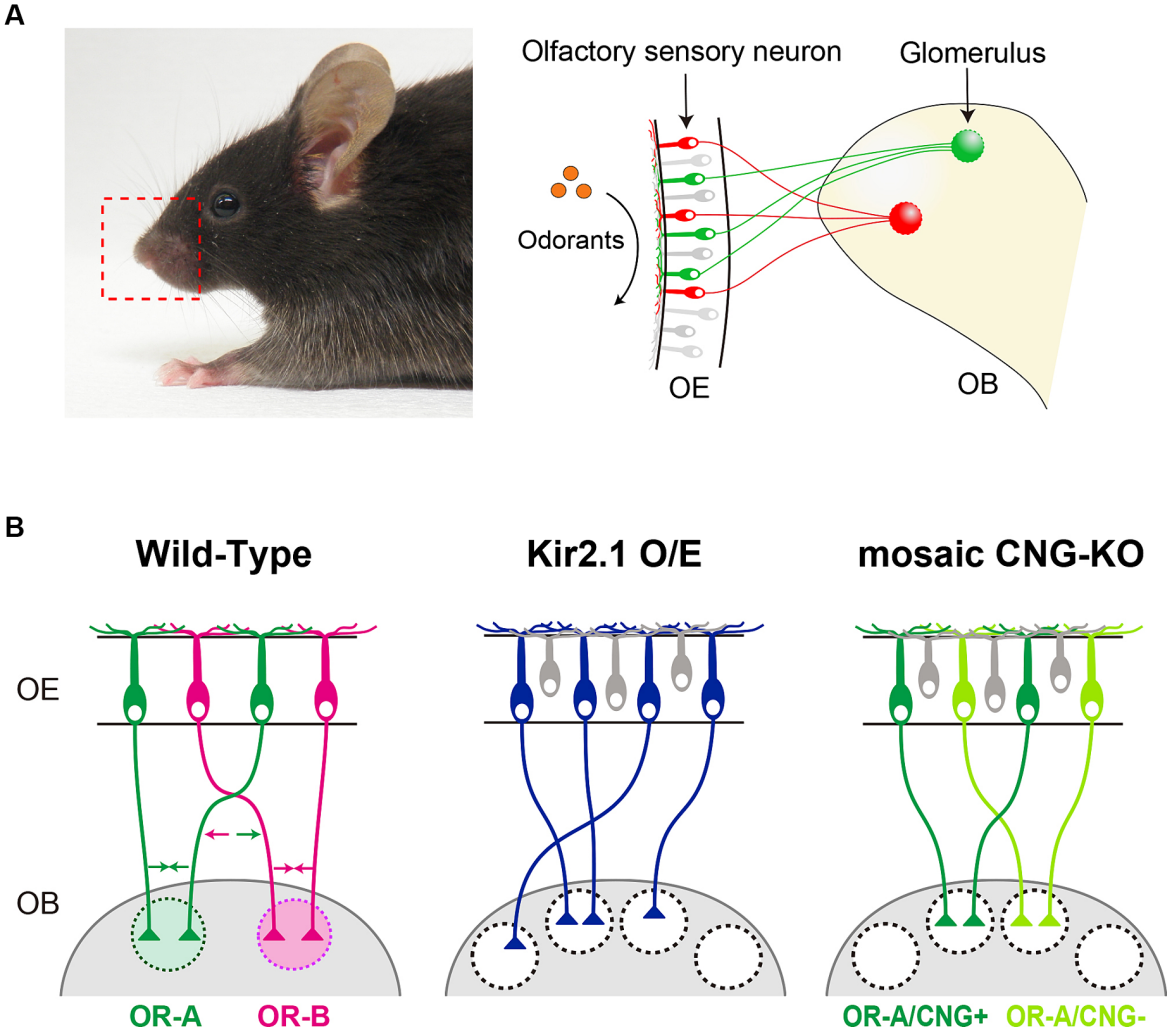
**(A)** In mice, each olfactory sensory neuron (OSN) in the olfactory epithelium (OE) exclusively expresses one type of odorant receptor (OR) gene. Axons from OSNs with the same OR converge at specific sites within the olfactory bulb (OB), forming glomerular structures. **(B)** Schematic diagrams illustrating the axonal projection of OSNs in wild-type mice, Kir2.1 overexpressing mice, and CNG channel mosaic KO mice. OSN axons expressing the same OR fasciculate into the same glomerulus by adhesive/repulsive interactions. Arrows in different colors indicate the adhesive or repulsive force of axon-sorting molecules (e.g., Kirrel2/3 and Eph/ephrinA-s) for convergence of like OSN axons.

Olfaction plays a crucial role in animal survival and reproduction in their natural environments, including food foraging, avoidance from predators, and social interactions. Odorants in the environment are detected by odorant receptors (ORs) that are expressed on olfactory sensory neurons (OSNs) within the olfactory epithelium (OE) of the nasal cavity (Buck and Axel, 1991). The binding of an odorant to ORs on cilia surfaces activates the G protein-adenylyl cyclase type III (ACIII) pathway, elevating cAMP levels, which subsequently opens cyclic nucleotide-gated (CNG) channels, resulting in the depolarization of OSNs (Breer et al., 1990; Bradley et al., 2005). In mice, ORs comprise the largest family of G protein-coupled receptors with >1,000 genes (Zhang and Firestein, 2002). Each OSN expresses only one functional OR gene, and axons from OSNs expressing a given OR converge onto a specific pair of glomeruli at stereotyped locations in the olfactory bulb (OB) (Mombaerts et al., 1996) (Figure 1A). Individual ORs can be activated by multiple odorants with differential sensitivities and vice versa, odorants activating multiple OR species (Malnic et al., 1999). Consequently, odor information is topographically represented as a pattern of activated glomeruli, generating the olfactory glomerular map in the OB.

During embryonic stages, an initial glomerular map is formed by a combination of axon guidance molecules whose expressions are defined by spatial cues and ligand-independent OR-derived basal activity (Sakano, 2010; Takeuchi and Sakano, 2014; Francia and Lodovichi, 2021; Dorrego-Rivas and Grubb, 2022). After OSN axons reach their approximate targets in the OB, further refinement of the glomerular map occurs, involving the fasciculation of similar OSN axons and the segregation of dissimilar ones. Like other sensory systems, neural activity is important for the refinement of the glomerular maps. Axonal convergence of OSNs is profoundly affected by genetic silencing of neural activity. In OSN-specific Kir2.1 overexpression mice where neural activity is blocked by a hyperpolarization of the membrane potential, OSN axons successfully project to the appropriate topographic location (Figure 1B) (Yu et al., 2004). However, they do not coalesce into specific glomeruli, which results in abnormally diffused glomeruli. Moreover, a mosaic knockout of the CNG channel, a critical element for generating odor-evoked neural activity, OSN axons expressing the same type of OR are segregated into distinct glomeruli that are innervated by either CNG-positive or CNG-negative axons (Figure 1B) (Serizawa et al., 2006). It has also been reported that hyperpolarization-activated cyclic nucleotide-gated (HCN) channels in olfactory sensory neurons regulate axon extension and glomerular formation (Mobley et al., 2010). In addition to neural activity, OR proteins have been shown to regulate the process of glomerular segregation with remarkable precision; just a single amino acid substitution of an OR sequence can generate distinct glomeruli in close proximity (Ishii et al., 2001; Feinstein and Mombaerts, 2004).

## 3 OR and neural activity-dependent glomerular formation

### 3.1 Molecular mechanisms of OR-dependent glomerular map refinement

Genetic experiments have demonstrated the roles of OR and neural activity in glomerular formation. The question arises as to whether these two processes independently regulate glomerular segregation or converge on a shared signaling pathway. To decipher the mechanisms underlying glomerular segregation, we began by dissecting the OR-dependent process at the molecular level. Contrary to the hypothesis that OR molecules serve merely as adhesive molecules due to their localization at the termini of OSN axons (Barnea et al., 2004; Strotmann et al., 2004), we explored the variations in gene expression profiles among OSNs expressing different ORs. In transgenic mice engineered to predominantly express a specific OR, we identified genes coding for homophilic adhesion molecules, such as Kirrel2 and Kirrel3 (Serizawa et al., 2006). These molecules are located at OSN axon termini and exhibited glomerular-specific and position-independent expression patterns at the OB. The expression levels of these molecules uniquely correlate with expressed OR types at the single-cell level. The mosaic gain or loss of function of these genes resulted in duplicated glomeruli even though the expressed ORs were the same, indicating that Kirrel2 and Kirrel3 are involved in the fasciculation process of like OSN axons. Notably, upregulated Kirrel2/3 levels in OSNs that naturally express these genes also led to duplicated glomeruli, highlighting that OSN axons discern not only the type but also the expression levels of axon sorting molecules (Serizawa et al., 2006). This quantitative difference in the expression level of these genes contributes to increasing variations in glomerular segregation. Additionally, repulsive molecules like ephrinAs and EphAs are expressed in an OR-specific and complementary manner across different OSN subsets (Serizawa et al., 2006). The interactions between subsets—one high in ephrinA and low in EphA, and vice versa—may be instrumental in segregating non-like OSN axons. Although the expression level of these molecules is correlated with OR types, their expression patterns are not identical (Ihara et al., 2016). Their unique expression profiles lead to the idea that a combinatorial code of adhesive and repulsive molecules imparts OR identity to OSN axons during glomerular formation. Notably, the expression of these molecules is affected by neural activity. For instance, a decrease in neural activity, such as the overexpression of Kir2.1 or CNG-KO, leads to opposite changes in their expressions: Kirrel2 and EphA5 expressions are downregulated by reduced neural activity, whereas Kirrel3 and ephrinA5 expressions are upregulated by reduced neural activity (Serizawa et al., 2006). These regulatory mechanisms of their expressions are consistent with the fact that glomerular segregation is OR-dependent and neural activity-dependent. OR-dependent and neural activity-dependent pathways in glomerular segregation converge in the expression pattern of axon sorting molecule. To date, several studies identified multiple activity-dependent axon-sorting molecules that are expressed in OSNs (St John et al., 2002; Cutforth et al., 2003; Kaneko-Goto et al., 2008; Williams et al., 2011; Ihara et al., 2016; Mountoufaris et al., 2017; Vaddadi et al., 2019; Wang et al., 2021; Martinez et al., 2023). The exact number of molecules implicated in this process is still unclear, but a handful of transmembrane proteins are thought to be involved. It is still not clear how much variation is produced by the combinatorial code model, and whether it produces differences comparable to the number of ORs. Future experiments will be needed to demonstrate how much difference in expression levels of a single axon sorting molecule can cause axonal segregation and whether these axon sorting molecules coordinately function in segregating axons as a combinatorial code.

### 3.2 Patterns of spontaneous activity link OR types and the molecular codes

As mentioned above, OR molecules control the expression of various axon-sorting molecules, which serve to regulate glomerular formation through their adhesive or repulsive interactions (Sakano, 2010; Takeuchi and Sakano, 2014; Francia and Lodovichi, 2021; Dorrego-Rivas and Grubb, 2022). The expression of these molecules is activity-dependent, consistent with the observation that glomerular formation is likewise activity-dependent. Since the Kir2.1 overexpressing mice exhibited a more severe phenotype compared to the CNG-KO, where mice are anosmia, spontaneous neural activity rather than odor-induced activity is more important for glomerular formation. It is anticipated that spontaneous neural activity serves as a bridge between OR types and the expression of axon-sorting molecules. Given the various expression patterns of axon sorting molecules and the numerous kinds of ORs, it is intriguing how neural activity links OR types to gene expression patterns of axon sorting molecules.

**Figure 2:**
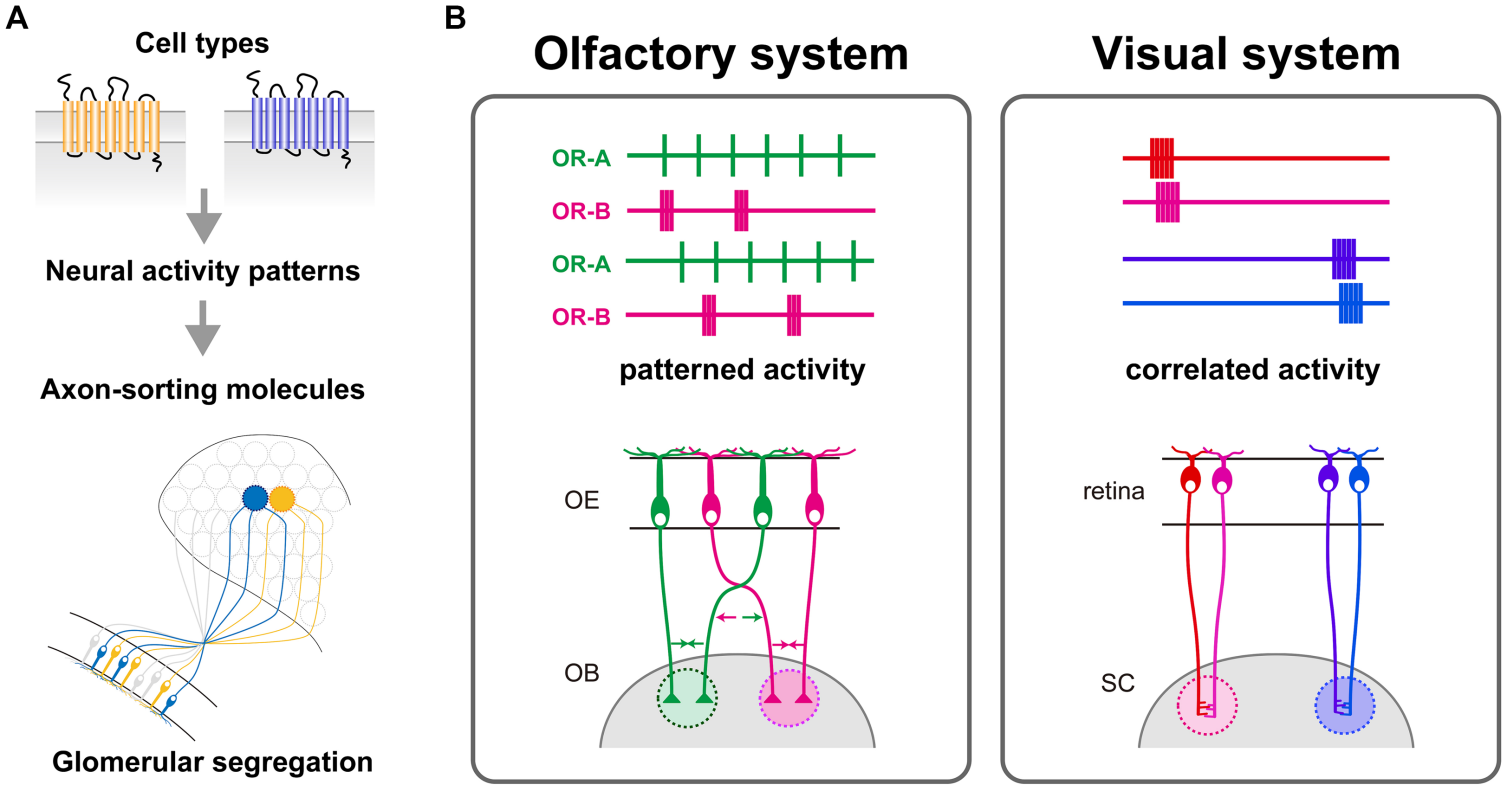
**(A)** OR-specific molecular codes, generated by differential expression of activity-dependent axon-sorting molecules at axon termini, regulate the glomerular segregation. These unique molecular codes arise from cell type-specific spontaneous activity patterns of OSNs, which are defined by the differences in expressed ORs. The conversion of OR-specific activity patterns into distinct combinations of axon-sorting molecules is crucial for the proper formation of the glomerular map. **(B)** Left: A model of activity-dependent refinement of the olfactory glomerular map. Temporal activity patterns reflect the identity of OSNs rather than the anatomical location; each color represents OSNs expressing the same OR, which converge into a common glomerulus. Right: A model of activity-dependent refinement of the retinotopic map in the visual system. Nearby retinal ganglion cells (RGCs) in the retina exhibit synchronized activity, and this correlated activity is crucial for establishing the spatially-organized topographic map in the superior colliculus (SC).

Through calcium imaging of OSNs, we have discerned that OSNs expressing different ORs elicit distinct temporal patterns of spontaneous neural activity (Nakashima et al., 2019). OR swap experiments revealed that the temporal patterns of spontaneous neural activity are regulated by the expressed ORs. Furthermore, optogenetically differentiated activity patterns induced specific expressions of corresponding axon-sorting molecules; for instance, short, high-frequency burst patterns particularly induced the expression of the axon-sorting molecule Kirrel2, whereas longer, lower-frequency bursts promoted the expression of other molecules like protocadherin10, also a participant in glomerular formation (Nakashima et al., 2019). Moreover, optogenetic stimulation resulted in the segregation of ChR2-positive glomeruli from those that were ChR2-negative, despite expressing the same OR. This phenomenon was accompanied by an upregulation of Kirrel2 proteins, indicating that artificially induced activity can overwrite the intrinsic OR identity. A significant breakthrough is the discovery that the unique temporal characteristics of spike patterns, rather than mere neural activity, provide the instructive signals for generating OR-specific expression profiles of axon-sorting molecules. This discovery proposes a novel activity-dependent mechanism, which is different from the prevailing Hebbian model of plasticity that requires correlated activity. In summary, this research unveils an activity-dependent mechanism where spontaneous spiking patterns instructively regulate the expression of axon guidance molecules, thus conferring OR identity to OSN axons for glomerular convergence (Figure 2A). However, it remains to be done how many variations there are in neural activity patterns of OSNs and how OSNs translate different temporal patterns of calcium dynamics into different gene expression patterns. To understand the intracellular mechanisms that translate neural activity patterns into gene expression patterns in OSNs, further analyses examining the expression and activity of neural activity-dependent transcriptional regulators in cells expressing different ORs are needed.

## 4 Discussion

It is widely accepted that neural activity is involved in mammalian neural circuit formation (Katz and Shatz, 1996; Spitzer, 2006; Luo and Flanagan, 2007; Sakano, 2010; Ackman and Crair, 2014; Pumo et al., 2022). The importance of neural activity in the formation of sensory maps has been demonstrated through pharmacological interventions or mutant mice that suppress neural activity (Kirkby et al., 2013; Antón-Bolaños et al., 2019; Martini et al., 2021). Recent advances in optogenetics have made it possible to control neural activity with higher temporal resolution and to elucidate the detailed role of neural activity in circuit formation (Adamantidis et al., 2015; Emiliani et al., 2022). For instance, in the visual system, asynchronous optogenetic activation of the left and right retinal ganglion cells resulted in normal eye-specific segregation in the superior colliculus and dorsal lateral geniculate nucleus, whereas synchronous activation disrupted eye-specific segregation (Zhang et al., 2011). Such optogenetic manipulations have significantly substantiated the significance of the Hebbian-based plasticity in sensory map formation (Figure 2B).

However, an activity-dependent process of the olfactory glomerular map seems not to follow the Hebbian rule (Figure 2B). Our findings demonstrated that there is no wave-like activity in the OE, which is observed in the developing retina. OSNs expressing the same OR do not fire simultaneously, but exhibit similar temporal patterns. Optogenetic stimulation with artificial activity patterns affected segregation of OSN axons, indicating the instructive role of temporal activity patterns in the olfactory circuit formation (Nakashima et al., 2019). Moreover, OSN axons fasciculate and form glomerulus-like structures even in mutant mice lacking synaptic partners (Bulfone et al., 1998; St John et al., 2003). Therefore, Hebbian theory, which postulates the simultaneous activation between pre- and post-synaptic neurons, is not suited for explaining the formation of the olfactory glomerular map. Thus, current studies support a model where spontaneous neural activity in OSNs plays a role in intracellular gene expression programs rather than interactions with other cells.

Since the discovery of the retinal wave (Galli and Maffei, 1988; Maffei and Galli-Resta, 1990; Meister et al., 1991), most models for activity-dependent neural map formation have been based on the assumption of synchronous activity (Figure 2B). Numerous experimental studies have provided evidence supporting the importance of synchronous activity in circuit development beyond sensory systems, such as the hippocampus and the neocortex (Ben-Ari et al., 1989; Garaschuk et al., 1998; Blankenship and Feller, 2010; Pan and Monje, 2020; Martini et al., 2021). While synchronous activity has been a dominant focus in studies of neural circuit formation, the findings obtained by olfactory circuit formation studies over the past few decades provide important insights that are not specific to the olfactory system but can be extended to other brain regions. Notably, analysis of cell type-specific spontaneous activity has revealed that precise temporal patterns, rather than synchrony *per se*, better reflect neuronal identity. Recent studies using single-cell RNA sequencing have uncovered distinct cell types at an unprecedented resolution (Saunders et al., 2018; Zeisel et al., 2018; Piwecka et al., 2023; Xing et al., 2023; Zhang et al., 2023). A key challenge moving forward will be to investigate whether cell type-specific temporal activity patterns are also present in other brain regions. Neural activity influences a multitude of developmental and plasticity processes, such as cell type specification, dendritic branching, synaptic maturation, and the underpinnings of learning and memory, through a complex program of gene regulation (Spitzer, 2006; Greer and Greenberg, 2008; West and Greenberg, 2011; Lee and Fields, 2021). However, their regulatory mechanisms are not fully understood. The breakthrough discovery in the olfactory system implicates the need to dissect the intricate spatiotemporal dynamics of neural activity to understand brain complexity at the molecular level.

## Author contributions

AN: Writing – original draft, Writing – review & editing. HT: Writing – original draft, Writing – review & editing.
